# Supplementary material for: Synthetic Design of Asymmetric miRNA with an Engineered 3′ Overhang to Improve Strand Selection
Source: Mol Ther Nucleic Acids. 2019 Apr 19;16:597–604. doi: 10.1016/j.omtn.2019.04.012 (PMC6517641; doi:10.1016/j.omtn.2019.04.012)
Supplement: Document S1. Figures S1–S3 [file mmc1.pdf]

OMTN, Volume *16*

Supplemental Information

Synthetic Design of Asymmetric miRNA with an Engineered 3′ Overhang to Improve Strand Selection

Sandeep Kadekar, Ganesh N. Nawale, Kira Karlsson, Cecilia Ålander, Oommen P. Oommen, and Oommen P. Varghese

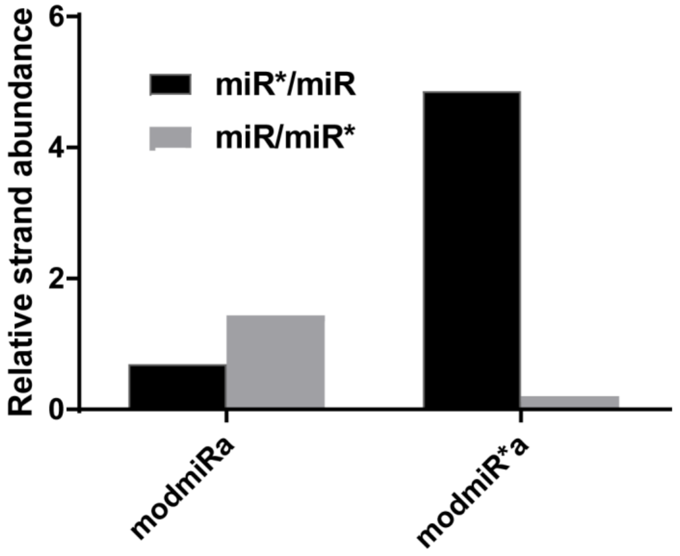

Figure S1. Stem loop qPCR analysis of strand recruitment for 5-nucleotide overhang miRNAs (modmiRa and modmiR*).

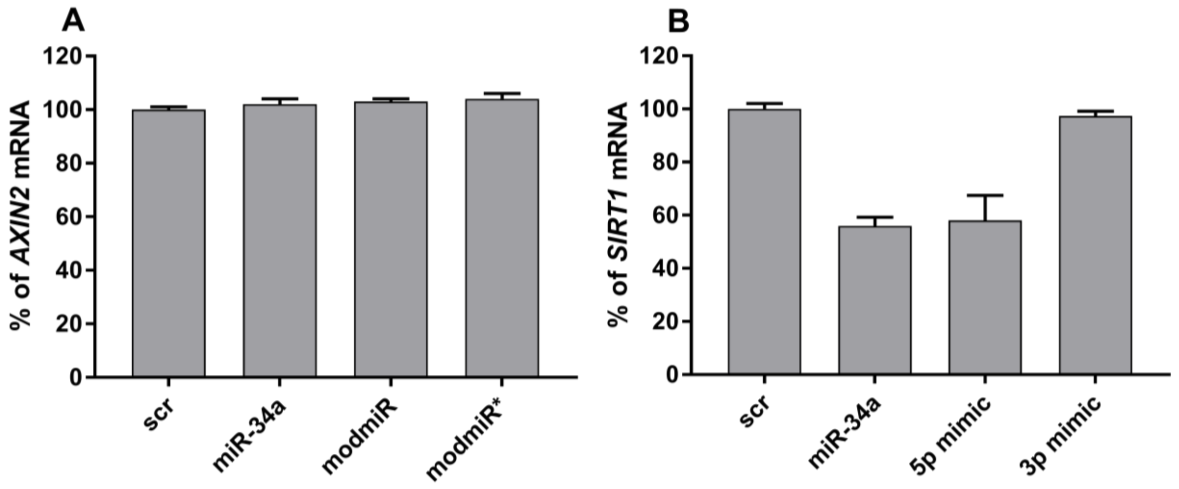

Figure S2. qPCR analysis for A) *AXIN2* and B) *SIRT1* mRNA levels. Cells were treated with different miRNAs for 48 h.

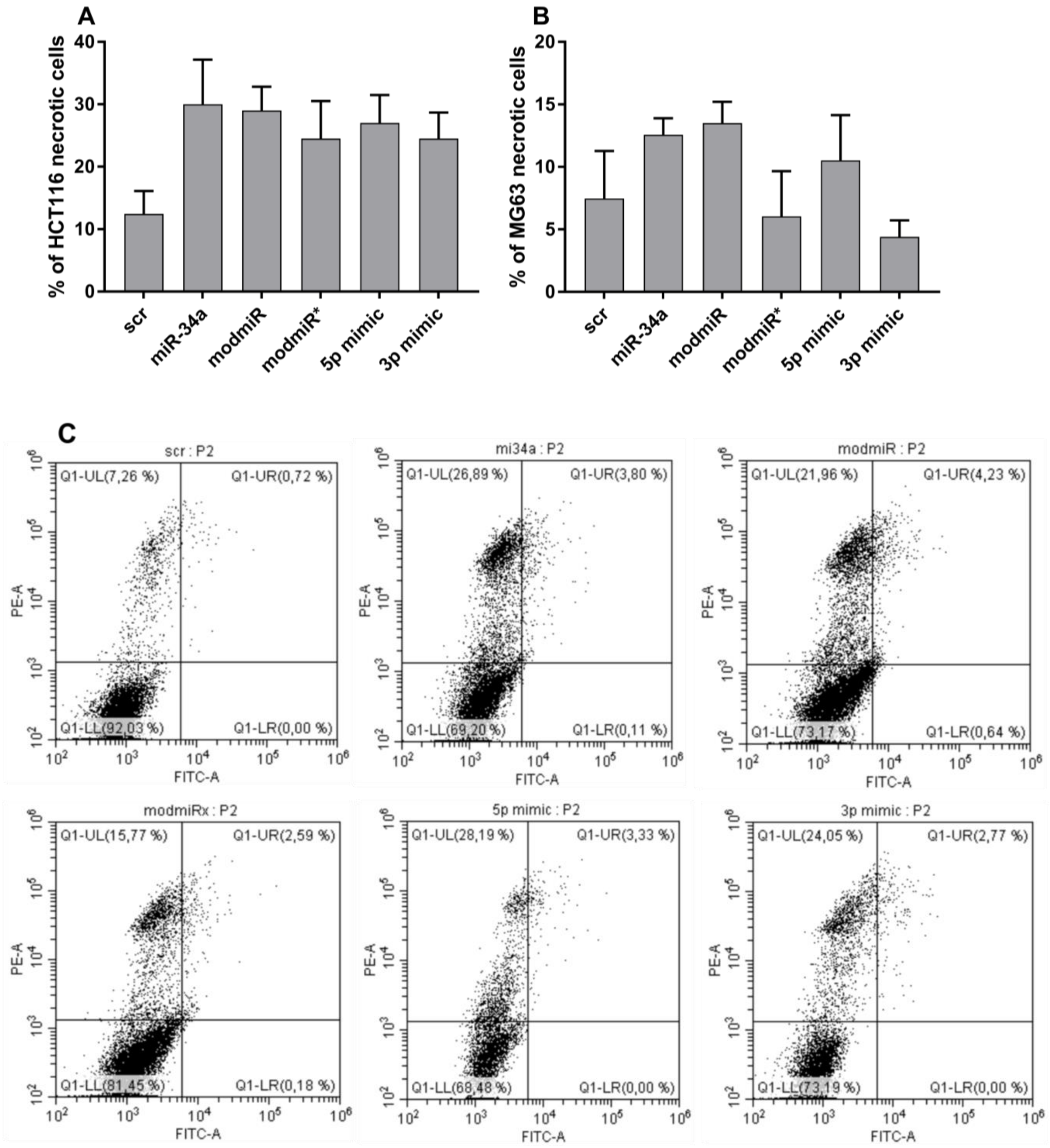

Figure S3. FACS analysis for HCT116 and MG63 cells. Cells were treated with different miRNAs for 48 h. A) and C) HCT116 cells were analysed for apoptotic and necrotic cells. B) MG63 cells were analysed for necrotic cells.
