# Supplementary material for: Synthetic Design of Asymmetric miRNA with an Engineered 3′ Overhang to Improve Strand Selection
Source: Mol Ther Nucleic Acids. 2019 Apr 19;16:597–604. doi: 10.1016/j.omtn.2019.04.012 (PMC6517641; doi:10.1016/j.omtn.2019.04.012)
Supplement: Document S2. Article plus Supplemental Information [file mmc2.pdf]

Molecular Therapy
Nucleic Acids
Original Article

Synthetic Design of Asymmetric miRNA with an Engineered 3′ Overhang to Improve Strand Selection

Sandeep Kadekar,[1] Ganesh N. Nawale,[1] Kira Karlsson,[1] Cecilia Ålander,[1] Oommen P. Oommen,[2] and Oommen P. Varghese[1]

[1]Translational Chemical Biology Laboratory, Polymer Chemistry Division, Department of Chemistry, Ångström Laboratory, Uppsala University, 751 21 Uppsala, Sweden; [2]Bioengineering and Nanomedicine Lab, Faculty of Medicine and Health Technology, Tampere University and BioMediTech Institute, 33720 Tampere, Finland

We developed a novel miRNA design that significantly improves strand selection within the RISC complex by engineering the 3′ end by adding extra nucleotides. Addition of seven nucleotides at the 3′ ends of the miR or miR* strand resulted in a thermodynamic asymmetry at either of the two ends, which resulted in selective RISC recruitment, as demonstrated by a stem-loop PCR experiment. Such selective recruitment was also corroborated at the protein level by western blot analysis. To investigate the functional effect because of selective recruitment, we performed apoptosis and metastasis studies using human colon carcinoma cells (HCT116) and human osteosarcoma cells (MG63). These experiments indicated that recruitment of the miR strand is responsible for inducing apoptosis and inhibiting the invasiveness of cancer cells. Recruitment of the miR* strand, on the other hand, had the opposite effect. To the best of our knowledge, our strand engineering strategy is the first report of improved strand selection of a desired miRNA strand by RISC without using any chemical modifications or mismatches. We believe that such structural modifications of miR34a could mitigate some of the off-target effects of miRNA therapy and would also allow a better understanding of sequence-specific gene regulation. Such a design could also be adapted to other miRNAs to enhance their therapeutic potential.

INTRODUCTION

MicroRNAs (miRNAs) belong to the large family of noncoding RNAs that regulate a variety of cellular process employing RNAi. These RNA molecules are formed from long single-stranded RNAs that are transcribed from genomic DNA.[1] miRNA was first identified in *C. elegans*, where it was found to regulate Lin− 14 mRNA through the 3′ UTR.[2] Endogenous miRNAs are found in almost all living systems, although their biogenesis involves different pathways in plants and animals. In mammals, miRNAs are transcribed by the RNA polymerase II gene and form hairpin loop structures that are processed by Drosha to form shorter double-stranded structures.[3] The shortened 21 to 22 nucleotide binds to mRNA and regulates the translation process or degrades the mRNA.[4] Unlike endogenous small interfering RNA (siRNA), miRNA duplexes have mismatches that allow unwinding of the duplex after binding to a ribonucleoprotein complex called RNA-induced silencing complex (RISC).[5] When the miRNA duplex binds to Argonaute (Ago) within the RISC, only one of the strands is fully incorporated, forming a mature RISC complex, whereas the other strand is discarded and degraded by nucleases in the cytosol.[6] The strand fully bound to RISC is used as a template to find mRNA targets, and most miRNAs have selectivity toward the 3′ UTR of the mRNA. Target recognition is determined by base-pairing between the seed sequence of the miRNA strand (2–8 nt from the 5′ end) and the mRNA transcript.[7] When a target mRNA is found, the RISC complex binds to the mRNA, blocking ribosomal binding, and recruits proteins that further induce translational repression.[8] Because only the short seed sequence is responsible for target binding, a single miRNA can target many sequences, regulating the expression of multiple genes.[7] Thus, dysregulation of miRNA expression can positively or negatively affect cellular processes.

One of the interesting features of endogenous miRNA expression is that it is indicative of biological processes both in healthy and disease states.[9] Thus, the therapeutic and diagnostic potential of miRNA will have major implications in future healthcare. One particular miRNA that has high therapeutic potential is miR-34a, which is currently investigated in phase I clinical trials.[10] Overexpression of miR-34a in cancer patients can downregulate the expression of more than 30 oncogenes as well as genes involved in tumor immune evasion.[11] These include genes involved in apoptosis, such as *SIRT1*, as well as those involved in metastasis, such as *MYC* and *CDK4*.[12] Downregulation of one of the targets of miR-34a, *SIRT1* leads to increased p53 expression, a tumor suppressor gene. Loss of miR-34a expression through hypermethylation of the miR-34a promoter region has led to progression in various cancer types. This suggests that miR-34a could

Received 15 November 2018; accepted 12 April 2019;
https://doi.org/10.1016/j.omtn.2019.04.012.

Correspondence: Oommen P. Varghese, Translational Chemical Biology Laboratory, Polymer Chemistry Division, Department of Chemistry, Ångström Laboratory, Uppsala University, 751 21 Uppsala, Sweden.
E-mail: oommen.varghese@kemi.uu.se

This is an open access article under the CC BY license (http://creativecommons.org/licenses/by/4.0/).

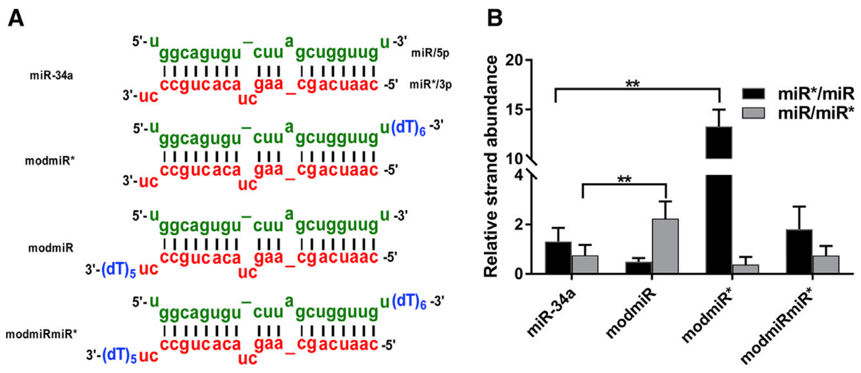

Figure 1. Design and Strand Selection of Modified miR-34a

(A) Structures of modified miR-34a. Green indicates the miR or 5p strand, whereas red indicates the miR* or 3p strand. The extra dT overhang is indicated in blue. (B) Stem-loop qPCR analysis of strand recruitment, displaying strand ratios between miR and miR* in HCT116 cells transfected with different miRNAs. Student's t test was used to determine statistical differences between pairs of groups. The difference in strand abundance between the indicated groups was significant (**$p \leq 0.01$).

be employed as an anticancer agent, slowing down metastasis and, if permissible, completely eliminating cancer phenotypes by reverting the cancer cells to preneoplastic conditions.[13]

To use miR-34a as a tool for anticancer therapy, all targets of miR-34a should be well defined. Unlike siRNA, which has a defined antisense strand, the two strands of miRNA, usually denoted as miR (5p) or miR* (3p), could participate in gene silencing activity, although the miR strand is generally known to play a major role. Because the entire potential binding sites for miR or miR* cannot be well defined within the transcriptome, it is imperative to tailor miRNA molecules with defined strand selection capability. Selection of the miR or miR* strand within the RISC complex is based on the orientation of the RNA duplex within the protein complex. It is generally believed that, similar to RISC loading of siRNA, miRNA loading is based on the thermodynamic asymmetry of the two ends of the duplex.[14] The strand having the less stable 5′ end is more likely to be selected as a guide strand, whereas the other strand, with the more stable 5′ end, serves as the passenger strand that is degraded. Although it is possible to design siRNA molecules with the desired asymmetry, it is not possible to optimize miRNAs because they are naturally identified from the biological milieu. In the case of miR-34a, it is known that both strands are biologically active and can target different genes.[15] Such dual strand activity could not only increase the therapeutic effect of the RNA molecule but could also impart undesired "off-target" effects that cannot be easily verified. In this article, we envisioned to develop modified miRNA with tailored thermodynamic asymmetry at the two ends of the duplex, which could modulate selective recruitment of the desired strand.

RESULTS

Design of Modified miR-34a and Stem-Loop qPCR Assay

To design asymmetric miRNAs, we incorporated extra deoxythymine nucleotides (dTs) at the 3′ end of both the miR (5p) and miR* (3p) strands (Figure 1A). Modifying the 3′ end is expected to influence duplex stability because it can destabilize the stacking interaction with the nearest neighbor.[16] To facilitate selective destabilization of the duplex to improve miR strand selection within the RISC complex, we added extra nucleotides at the 3′ end of the miR* strand. To reverse the strand selection and favor miR* strand selection, the extra nucleotides were added to the 3′ end of the miR strand (Figure 1A). Because natural miR-34a has a 2-nt overhang at the 3′ end on the miR* strand, we increased the overhang length to 5 and 7 nt by adding 3 and 5 dT nucleotides, respectively. Because the 3′ end of miR strand of miR-34a has a single nucleotide overhang, we increased the overhang length to five and seven by adding four and six dT nucleotides, respectively (Figure 1A).

To evaluate the effect of the modification on strand selection, we performed transfection experiments in a human colon carcinoma cell line (HCT116). Because it is known that the recruitment of a particular strand within the RISC increases the cytosolic concentration of that specific strand, we determined the levels of each strand by stem-loop qPCR experiments.[11] Quantitative determination of each strand after transfection gave a very surprising result. The natural miR-34a demonstrated equal propensity to recruit either strand. However, the 5-nt modification did not show a significant difference when used for selection of miR compared with miR-34a, but this modification resulted in an increase in miR* strand concentration (Figure S1). Incorporation of the 7-nt overhang, on the other hand, clearly indicated a bias for one strand or the other strand as a result of structural modification (Figure 1B). We therefore termed modified double-stranded miR-34a with selectivity for miR or the guide strand "modmiR" (5p selection), whereas the sequence that showed selectivity for the miR* strand was termed "modmiR*" (3p selection). We also designed dual strand-modified miR-34a with 7-nt overhangs on both the miR and miR* strands (termed "modmiRmiR*") (Figure 1A). To determine the increase of a specific strand recruitment, we estimated the relative ratio of miR and miR* strand employing a stem-loop qPCR assay, as mentioned above (Figure 1B). The unmodified miR-34a indicated a ratio of close to one, suggesting a similar abundance of both strands in the cytosol (Figure 1B). The symmetrical dual-modified modmiRmiR* miRNA showed a pattern almost identical to the miR-34a experiment. Interestingly, modmiR* variants of miR-34a showed a significant increase in the miR* strand (13-fold increase). The modmiR variant also had the anticipated strand bias, with a higher concentration of the miR strand compared with the miR* strand (2.2-fold increase). Taken together, our stem-loop qPCR results reinforce the idea of

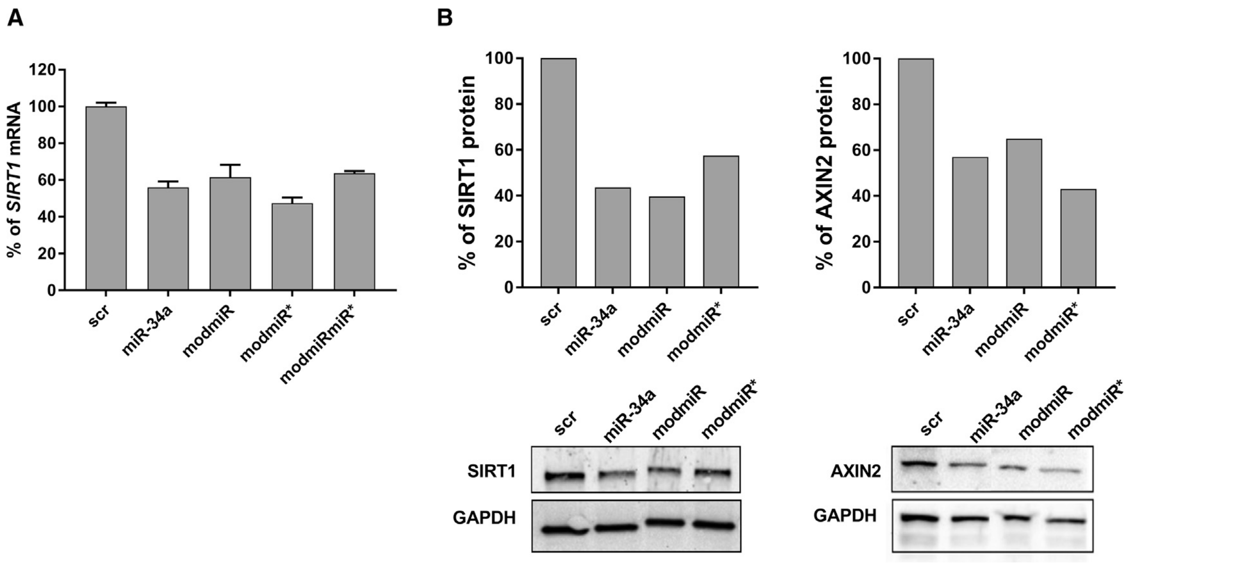

Figure 2. Effect of Different miR-34a on Gene Knockdown at the mRNA and Protein Levels

(A) HCT116 cells were transfected with different miRNAs for 48 h and analyzed by qPCR for *SIRT1* mRNA levels. (B) HCT116 cells were transfected with different miRNAs for 72 h for *SIRT1* protein levels and for 96 h for *AXIN2* protein levels by western blotting. Densitometry on protein bands was done using ImageJ.

modified miRNAs displaying strand selectivity, which could lead to a difference in functional activity.

Strand Selection at the mRNA and Protein Levels

To determine functional effects as a result of strand selection, we analyzed the known targets of miR-34a. One such target of miR-34a is *SIRT1*, which is reported to be specific for the miR (miR-34a-5p) strand,[17] whereas other targets, such as *AXIN2*, are known to be specific for the miR* (miR-34a-3p) strand. Transfection of differently modified miR-34a analogs (modmiR, modmiR*, and modmiRmiR*) and evaluation of the mRNA levels of *SIRT1* (Figure 2A) and *AXIN2* (Figure S2A) by qPCR did not show any significant difference in gene knockdown compared with the unmodified miR-34a. Such a marginal effect of strand selection could be due to the accumulation of targeted mRNA in p bodies because they are not generally degraded.[6] We therefore, investigated the effect of gene knockdown at the protein levels using western blot assays. Western blot analyses were performed at different time points post-transfection to observe the differences in protein levels between cells treated with differently modified miR-34a. These experiments suggested that the 72-h time point is suitable for *SIRT1* knockdown, whereas 96 h was found to be optimal for *AXIN2*. The analysis of immunoblots indicated distinct bands of *SIRT1* and *AXIN2* (Figure 2B) proteins with clear differences in density between groups. Densitometric analysis of the plot indicated that approximately 57% of the *SIRT1* protein was silenced using conventional miR-34a compared with the negative control (scrambled sequence [scr]). The transfection experiment with modmiR indicated a similar level of protein knockdown as miR-34a (~61%), whereas modmiR* demonstrated a lower level of knockdown (~40%). These results suggest that strand bias as a result of miRNA modification indeed modulates protein levels to some extent. Investigation of *AXIN2*, on the other hand, indicated that miR-34a induces silencing of *AXIN2* by ~43%, whereas modmiR reduced the protein levels by ~35%. Interestingly, modmiR* reduced the protein target by ~57%, which was anticipated because it is specific for the miR* target (Figure 2B). Overall, the western blot results indicated that structural modification of miR-34a could be reflected at the protein level, which correlates with the strand selection phenomenon observed by stem-loop PCR experiments.

Apoptosis Assay

Intrigued by the target-specific gene knockdown because of our structural design, we decided to explore the therapeutic potential of the modified miRNA sequences. For this purpose, we investigated the effect of modified miRNA sequences on apoptosis or necrosis of cancer cells and compared it with miR-34a. For this purpose, we performed an apoptosis assay on HCT116 cells as well as on MG63, a human osteosarcoma cell line. Because apoptosis of cells results in membrane disruption, exposing the intramembrane phosphatidylserine sites on the membrane surface,[18] labeling of this lipid with fluorescently labeled Annexin V has been suggested to provide direct evidence of programmed cell death. Flow cytometry analysis of HCT116 cells 48 h after transfection with different miRNA analogs indicated that miR-34a, along with modmiR, increased the apoptosis level, as reported earlier, by approximately 4% (Figure 3A).[19] The number of apoptotic cells was reduced by 2% when transfection was performed with modmiR* compared with miR-34a, which was found to be statistically significant. Earlier studies have shown that a commercially available 5p mimic (a chemically modified double-stranded RNA designed to supplement 5p strand-specific miRNA activity) of miR-34a promotes apoptosis of cancer cells.[17] We therefore compared the effect of the 5p mimic with that of modmiR in the apoptosis assay. Our

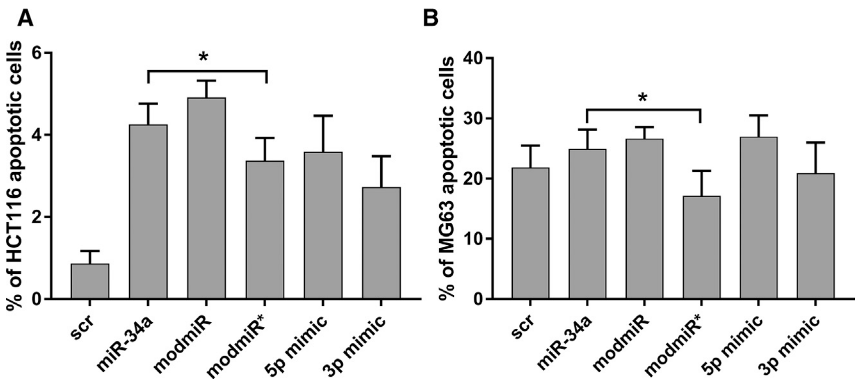

Figure 3. Apoptosis Assay of Cells Treated with Different miR-34a

(A and B) HCT116 cells (A) and MG63 cells (B) were transfected with 25 nM of different miR-34a for 48 h and analyzed by fluorescence-activated cell sorting (FACS) for apoptotic cells. Annexin V-FITC dye was used for labeling of apoptotic cells. Student's t test was used to determine statistical differences between pairs of groups. The difference in apoptotic cells between the indicated groups was significant (*$p \leq 0.05$).

results clearly show that modmiR yielded a better apoptotic effect compared with the 5p mimic and miR-34a. We also compared the effect of the commercial 3p mimic with that of modmiR*, which indicated a similar level of apoptotic cells, indicating that the miR strand is responsible for apoptosis. Of note, we observed no significant difference in apoptotic cells in the 5p mimic group compared with miR-34a. We also observed necrosis when the cells were treated with modified miR-34a in a similar pattern as observed in apoptosis (Figures S3A and S3C). When we performed the apoptosis assay with MG63 cells using modified miR-34a, we observed similar apoptosis (Figure 3B) and necrosis (Figure S3B) effects as observed with HCT116 cells. As anticipated, we observed a significant drop in apoptosis with modmiR* compared with miR-34a.

Invasion Assay

Cancer cell invasion is a process by which cancer cells invade their extracellular matrix environment (basement membrane and stroma) to reach the blood stream and, eventually, other organs.[20] Interestingly, miR-34a is known to inhibit cancer cell invasion of HCT116 cells by increasing apoptosis.[17] We therefore investigated the effect of our modifications to inhibit cancer cell invasion using Matrigel (a mouse tumor-derived extracellular matrix that mimics the tumor microenvironment).[21] The cells were treated with different miR-34a, and invasion activity was analyzed by counting the number of cells on the basal surface of the Transwell after 48 h of treatment (Figure 4). As anticipated, miR-34a significantly reduced the number of invasive cells compared with cells treated with the negative control (scrambled sequence) (Figure 4). Interestingly, cells treated with modmiR exhibited marginally fewer invasive cells compared with miR-34a-treated cells, whereas modmiR* had a higher number of invasive cells, similar to negative control-treated cells. We further compared the invasiveness of modified miR-34a-treated cells with commercially available 5p and 3p mimics. These experiments indicated that cells treated with the 5p or 3p mimic had a similar effect as unmodified miR-34a. In summary, the invasion assay result corroborates the apoptosis experiments, indicating a role of the miR strand in inducing the anticancer effects of miR-34a. Thus, strategies that could improve selective recruitment of the miR strand could be beneficial for anticancer therapy.

DISCUSSION

Synthetic miRNA mimics that could modulate gene expression similar to endogenous miRNA have enormous potential as therapeutic agents for treating a variety of human conditions from cardiovascular diseases[22] to cancer,[9] provided there is an efficient cell-specific delivery method. We have recently shown that native extracellular matrix-derived biopolymers can promote intracellular delivery of nucleic acids, exploiting hydrophobic interactions.[23] Unlike endogenous gene regulation by natural miRNA, the effects of synthetic mimics on gene regulation significantly depend on the concentration of the miRNA used or whether it is expressed using a plasmid or viral vector.[24] Such mimics could also increase off-target effects by increased loading of the passenger strand within the RISC.[25] Thus, strategies that can promote selective recruitment of the desired functional strand could significantly reduce off-target effects as well as enable effective gene regulation at a lower concentration of the mimics. This prompted us to develop an asymmetric miRNA design that would allow selective recruitment of the desired strand within the RISC. To design asymmetric miRNA, we selected a therapeutic miRNA (miR-34a) and incorporated 5- to 7-nt overhangs at the 3′ end. Extension of the 3′ end naturally occurs in many matured miRNAs with the help of terminal nucleotidyl transferases that preferentially introduce uridyl or adenyl residues to the 3′ end.[26] It is generally believed that such an extension, even though improves intracellular stability, renders miRNA biologically inactive because it affects RISC loading of the matured miRNA. We therefore decided to evaluate whether this is indeed true for a synthetic mimic and added three or five dT units at the 3′ end of the miR* strand and four or six dT units at the 3′ end of the miR strand to obtain an overhang length of 5–7 nt. Our rationale for such an extension of 3′ overhangs was to incorporate a "wagging tail" that could result in selective destabilization of the duplex at one or the other end of the mature miRNA. Because RISC loading of the miR or miR* sequences is believed to be dependent on the thermodynamic asymmetry principle,[14] we anticipated that such selective destabilization should render selective loading of the miR or miR* strands.

To test our hypothesis of selective recruitment of the miR or miR* strands, we chose miR-34a as a model miRNA because it is one of the well-studied miRNAs that are known to be involved in different

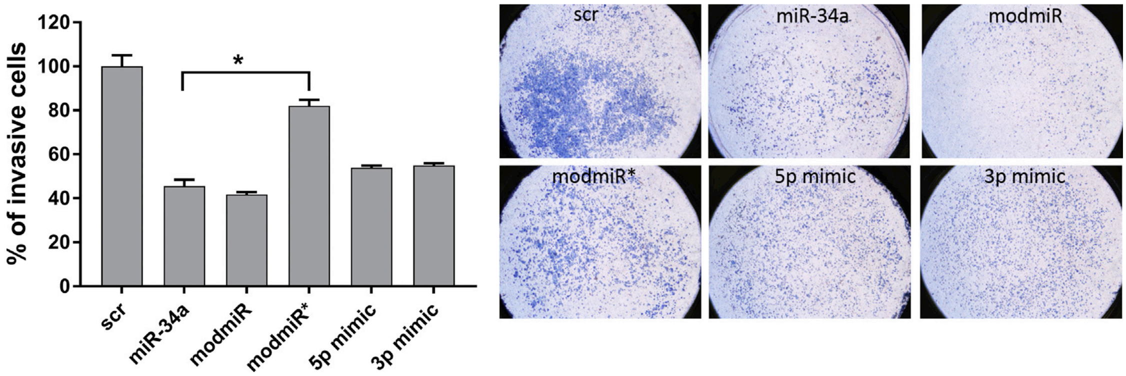

Figure 4. Effect of miRNAs on Invasion of HCT116 Cells

Cells were transfected with different miRNAs for 24 h and seeded for an invasion assay for 48 h, followed by fixing and staining of invasive cells. The graph indicates the percentage of invasive cells. Student's t test was used to determine statistical differences between pairs of groups. The difference in invasive cells between the indicated groups was significant (*$p \leq 0.05$).

types of cancer,[10,17,27] osteoporosis, and bone metastasis[28] as well as in cardiovascular diseases.[22] Interestingly, some studies with miR-34a also indicated dual strand activity, which would allow us to quantify the role of miR or miR* strands separately. Of note, there are few commercially available mimics of miRNA (e.g., a miRNA mimic from Thermo Fisher Scientific and miRIDIAN from Dharmacon), which are chemically modified double-stranded miRNAs that show selectivity for miR or miR* strands. However, the exact chemical structures of these mimics are not reported, and to the best of our knowledge, there are no non-chemically modified miRNAs that show strand selectivity. Our study represents the first report where strand activity of a miRNA could be regulated without any chemical modifications. Our structural designs of modmiR and modmiR* were able to induce thermodynamic asymmetry, which could be reflected in stem-loop qPCR experiments, indicating selective strand selection within the RISC. These results were further corroborated using western blot analysis, which provided direct evidence for strand selection at the protein level (*SIRT1*, an miR target, and *AXIN2*, an miR* target). The effect of strand selection was also reflected in the functional analysis, as determined using apoptosis and invasion assays in two different cancer cells. These experiments indicated that the miR strand is responsible for inducing apoptosis and for inhibiting invasion, which could be improved by our structural design. Selection of the miR* strand, on the other hand, had an opposite effect. These results clearly suggest that miRNA modifications that have specific strand selection capability could modulate the anticancer effect of miR-34a. Of note, our modified miRNA had a better therapeutic effect compared with the commercially available mimics. Because we employed natural nucleotides for modifying miRNA, it would also limit any undesired toxicity that may arise from modified nucleotides generally used in commercial mimics. Such a design could be adapted to other clinically relevant miRNAs, which could address some of the off-target effects associated with the recruitment of undesired strands. We believe that our design strategy will also open new avenues for researchers to study individual strand activity of different miRNAs.

MATERIALS AND METHODS

miRNA Synthesis

The sequence for miR-34a was obtained from miRBase. Unmodified phosphoramidites and solid support were purchased from ChemGene (USA). All reagents used for RNA solid-phase synthesis were purchased from Sigma-Aldrich (Sweden). Lambda 35 UV-visible (UV-vis) spectrophotometer from PerkinElmer was used for spectroscopic analysis. miRNA sequences were synthesized using an automated solid-phase synthesizer (H8, K&A synthesizer) with 2′-O-TBDMS-protected monomers employing a standard synthesis cycle using phosphoramidite chemistry. Cleavage from support and deprotection of base-protecting groups was carried out by treating the beads with ammonia and methylamine (AMA) solution (1 mL, 41% methylamine in water and 30% aqueous [aq.] NH_3 [1:1 v/v]). The 2′-O-TBDMS groups were deprotected using Et3N·3HF in DMSO. RNA was purified by 20% denaturing PAGE (7 M urea) and recovered with Tris-EDTA-NaCl (TEN) buffer. The RNA samples were desalted using a Sep-Pak (WAT020515, Waters) column. The pure RNA pellet was dissolved in water, and the concentration was measured at 260 nm in a UV-vis spectrophotometer. Hybridization into the duplexes of different miR-34a sequences (Figure 1A) was prepared using equimolar concentrations of the respective complementary strands in water. The solution containing both strands was heated at 95°C for 2 min and then gradually cooled to room temperature over a period of 3 h. Mimics for miR-34a, 3p, and 5p were procured from Thermo Fisher Scientific (Sweden).

Cell Transfections

HCT116 (human colon cancer) cells and MG63 (human osteosarcoma cells) were obtained from the ATCC (Manassas, VA, USA). A day prior to transfection, HCT116 cells were seeded at a density of 100,000 cells/mL in a 12-well cell culture plate to achieve ~60%–80% confluence at the time of transfection. DMEM consisting of high glucose, 10% fetal bovine serum (FBS), and 1% antibiotics (penicillin streptomycin [PeSt]; Thermo Fisher Scientific, Sweden) at 37°C, and 5% CO_2 was used as a medium. On the day of

transfection, the medium was replaced with fresh complete DMEM. The miRNAs (unmodified and modified; Figure 1A) were transfected with 100-nM concentrations employing RNAiMAX transfection reagent (Thermo Fisher Scientific, Sweden) according to the manufacturer's protocols. Briefly, the miRNA was mixed with RNAiMAX reagent and incubated for 5 min. This complex was subsequently added to the cells to be transfected. Cells were also transfected with negative control siRNA (scrambled sequence). Each transfection was performed in duplicate. Post-transfection, cells were incubated for 24 h.

Total RNA Samples

After incubation as stated above, total RNA was isolated from cells by adding 350 μL of lysis buffer (QIAGEN, Germany), followed by homogenization of cell lysates. RNA was extracted from cell lysates using the RNeasy Mini Kit (QIAGEN, Germany). The NanoDrop 2000 (Thermo Fisher Scientific, Sweden) was used to determine RNA concentrations, with resulting optical density (OD) 260/280 ratios between 1.85–2.03.

Real-Time qPCR

1 μg of the total RNA was used to make the cDNA. The cDNA was prepared using the High Capacity RNA to cDNA kit according to the manufacturer's protocol (Applied Biosystems, USA), and real-time qPCR was performed with cDNA and TaqMan Fast Universal PCR Master Mix (2X) (Applied Biosystems, USA). The real-time PCR reactions were carried out with 10 μL of 2x TaqMan Universal PCR Master Mix, no AmpErase uracil-N-glycosylase (UNG) (Applied Biosystems, USA), 5 μL diluted cDNA, and 1 μL of TaqMan gene-specific assay mix (Applied Biosystems, USA) in a 20-μL final reaction volume. The reference gene β-actin (*ACTB*) and the sample genes *SIRT1* and *AXIN2* (TaqMan primers, Thermo Fisher Scientific, Sweden) were selected as controls for normalization of real-time PCR data. Amplification was carried out using the CFX Connect System (Bio-Rad, Sweden) using a 40-cycle program. The CFX Manager software automatically calculates the raw Ct (cycle threshold) values. Data from samples with a Ct value equal to or below 30 were analyzed further. Samples were normalized relative to the endogenous control, and differences in cycle number thresholds were calculated using the comparative quantitation $2^{-\Delta\Delta CT}$ method (also called the ΔΔCT method), which is commonly used for analyzing siRNA-induced gene knockdown efficiency.

The formulas used to calculate gene knockdown were as follows. First, the ΔCT was calculated as the mean cycle threshold for the target gene minus the mean cycle thresholds for the endogenous controls *ACTB*, each performed in triplicate: ΔCT = CT (target gene) – CT (endogenous control). Second, the ΔΔCT was calculated as the ΔCT of the target minus the ΔCT of the negative control (NC): ΔΔCT = ΔCT (target) – ΔCT (NC). Thereafter, the percentage of knockdown of the target gene was calculated as follows: Fold change = 2−ΔΔCT, then percentage of knockdown: = 100 * (1-fold change).

Stem-Loop qPCR

For stem-loop qPCR, similar transfection conditions and amounts as for real-time PCR were used for transfection. After transfection, RNA was isolated using the Mirvana miRNA isolation kit (Thermo Fisher Scientific, Sweden) following the manufacturer's protocol. Stem-loop primers were used to make cDNA by using 10 ng of RNA. Stem-loop primers and qPCR TaqMan primers specific for each strand of miR-34a and miR-21 (control gene) were custom-ordered (Thermo Fisher Scientific, Sweden). cDNA synthesis was done in accordance with the manufacturer's protocol. Real-time PCR was done in the same manner as described in the previous section. The SEM of all primer ratios was used to determine statistical variation. A Student's t test was performed to evaluate significance, with p values noted as follows: $^{*}p \leq 0.05$, $^{**}p \leq 0.01$, and $^{***}p \leq 0.001$.

Western Blot Assays

Primary antibodies for the miR-34a targets *SIRT1* (Santa Cruz Biotechnology, USA) and *AXIN2* (Cell Signaling Technology, USA) and the control protein glyceraldehyde-3-phosphate dehydrogenase (*GAPDH*) (Cell Signaling Technology, USA), were procured from Abcam. HCT116 cells were seeded in 6-well plates with 200,000 cells in 2 mL DMEM per well. Transfection was performed as described for stem-loop qPCR with 100 nM miRNA, and the cells were incubated for 72 h for *SIRT1* and 96 h for *AXIN2*. Thereafter, the cells were washed twice with PBS (Thermo Fisher Scientific, Sweden), and 200 μL lysis buffer (Pierce radioimmunoprecipitation assay [RIPA] buffer with 1% protease inhibitors; Thermo Fisher Scientific, Sweden) was added to the wells, followed by scraping using cell scrapers. The lysates were collected in microcentrifuge tubes, flash-frozen in liquid nitrogen, and stored at −20°C. Protein concentration was determined using the Bradford protein assay (Thermo Fisher Scientific, Sweden) by creating a standard curve of BSA with known concentrations of protein. The BSA protein was diluted to concentrations ranging from 0 to 2 mg/mL, and 4 μL of each concentration was incubated with 100 μL Pierce Coomassie Plus (Bradford) assay reagent (Thermo Fisher, Sweden) in a 96-well plate at room temperature for 10 min. Absorbance was measured at 595 nm using a Tecan Infinite M200 microplate reader (Tecan, Switzerland), and a standard curve with absorbance as a function of protein concentration was set up using the data points. The protein samples were centrifuged in a Himac CT15RE tabletop centrifuge (Hitachi Koki) for 15 min at 15,000 rpm, and absorbance was determined using the method described for BSA. The protein concentration of the samples was determined using the linear correlation from the standard curve. To prepare samples for SDS-PAGE, equal quantities of protein (ranging between 15–30 mg depending on the lowest protein concentration) from the samples were mixed with water and loading dye (4× Laemmli buffer containing 10% β-mercaptoethanol; Bio-Rad, Sweden) to a total volume of 60 μL in microcentrifuge tubes. The samples were denatured at 95°C for 5 min and centrifuged briefly. The Mini-PROTEAN Tetra cell System (Bio-Rad, Sweden) was set up using Mini-PROTEAN TGX precast gels (Bio-Rad, Sweden). 20 μL of each protein sample was loaded onto the gel. The Tetra cell system was run at 100 V for approximately 40 min until the protein bands reach the bottom of the gel. The protein bands were transferred from the gel onto a polyvinylidene fluoride (PVDF) (Bio-Rad, Sweden) blot using the Trans-Blot Turbo Transfer System (Bio-Rad,

Sweden). The blot was incubated in blocking solution (1× PBS with 1% casein; Bio-Rad, Sweden) with gentle rotation at room temperature for 1 h and cut into two pieces to accommodate incubation with two different antibodies. The blot pieces were placed in blocking solution containing primary rabbit antibodies specific to *SIRT1* and *AXIN2* diluted 1:100 and *GAPDH* diluted 1:5,000, respectively, and incubated for 24 h at 4°C. The next day, the blots were washed in TBST (1% Tween 1× Tris-buffered saline buffer; Bio-Rad, Sweden) with gentle rotation for 1 h, changing the buffer every 15 min, followed by incubation in secondary goat anti-rabbit antibodies diluted 1:2,000 for *SIRT1* and *AXIN2* and 1:5,000 for *GAPDH* for 1 h. Finally, the blots were incubated in 4 mL visualization solution from Clarity (Bio-Rad, Sweden) and taken for camera exposure using a Molecular Imager Gel Doc XR System (Bio-Rad, Sweden). Densitometry of the bands obtained from visualization of the bands was done by ImageJ.

Flow Cytometry

Different concentrations and time points were tried to arrive at the concentration and time point that showed the highest difference among the miRNAs. After testing different concentrations, 25 nM and 48 h incubation time were selected because they showed a significant difference. HCT116 and MG63 cells were grown in 12-well plates with 100,000 cells per well and incubated for 24 h. After 24 h, the cells were transfected with 25 nM miRNA using the conditions described for stem-loop qPCR and incubated for 48 h at 37°C. The cells were washed with PBS and detached using 200 μL TrypLE (Thermo Fisher Scientific, Sweden) for each well. Following the instructions of the Annexin V-FITC Apoptosis Staining/Detection Kit (ab14085, Abcam, USA), cells were resuspended in 500 μL assay buffer, and 5 μL of Annexin V with the fluorescent dye fluorescein isothiocyanate (FITC) and 5 μL propidium iodide were added. Cells were then incubated at room temperature in darkness for 5 min. The cells were measured using a CytoFLEX S Research Flow Cytometer (Beckman Coulter, USA) with the flow rate set at medium level. Gain settings utilized for measurement were as follows: FITC, 10; phycoerythrin (PE), 10; side scatter or forward scatter, 20. Around 10,000 events were recorded, and the data were analyzed using the software Kaluza (Beckman Coulter, USA). Single cells were analyzed, and doublets were excluded by using FSC-A-FSC-H plots. Untreated cells were used as controls, and all treated cells were compared with the untreated cells. Percentages of cells with Annexin FITC and propidium iodide were selected for analysis to determine apoptotic and necrotic cells.

Invasion Assay

Cells were grown in 12-well plates with 100,000 cells per well and incubated for 24 h. The next day, cells were transfected with 100 nM miRNA using the conditions described for stem-loop qPCR and incubated for 24 h at 37°C. Corning BioCoat Matrigel Invasion Chambers (Corning Life Sciences, USA) were used for the assays. The inserts were incubated with cell medium for HCT116 cells without FBS and PeSt in the bottom and the top for 2 h at 37°C and 5% CO_2. Further, pre-transfected HCT116 cells were detached from the cell culture plate with TrypLE (Thermo Fisher Scientific, Sweden) and counted. Thereafter, 100,000 cells were diluted with medium without FBS and PeSt and added to the top part of the insert while medium with FBS and PeSt was added to the bottom part of the insert and incubated for 48 h. After incubation, the cells in the top part of the insert were removed, and the cells in the bottom part of the insert were fixed with methanol for 2 min, stained with 0.2% toluidine blue in water (Sigma-Aldrich, Sweden) for 10 min, and then washed 4 times with water for 10 min. After washing, the inserts were air-dried, imaged further, and counted under a microscope (2×).

Statistical Analysis

Student's t test was used to determine statistical differences between pairs of groups. $p < 0.05$ and $p < 0.01$ (two-sided) was considered statistically significant. Data were analyzed using the GraphPad Prism software package (version 7.0).

SUPPLEMENTAL INFORMATION

Supplemental Information can be found online at https://doi.org/10.1016/j.omtn.2019.04.012.

AUTHOR CONTRIBUTIONS

S.K., G.N.N., C.A., and K.K. performed all experiments. O.P.V. conceived the project and designed the experiments. O.P.O. and O.P.V. participated in critical analysis of the data. All authors contributed to writing of the manuscript.

CONFLICTS OF INTEREST

O.P.O. and O.P.V. are part of Uppsala Therapeutics AB.

ACKNOWLEDGMENTS

We would like to thank the Biovis facility (Uppsala University) for help with the FACS experiments. We acknowledge financial support from Swedish Strategic Research "StemTherapy" (Dnr 2009-1035) and the Swedish Foundation for Strategic Research (SSF, SBE13-0028).

REFERENCES

1. Ha, M., and Kim, V.N. (2014). Regulation of microRNA biogenesis. Nat. Rev. Mol. Cell Biol. *15*, 509–524.
2. Lee, R.C., Feinbaum, R.L., and Ambros, V. (1993). The C. elegans heterochronic gene lin-4 encodes small RNAs with antisense complementarity to lin-14. Cell *75*, 843–854.
3. Schanen, B.C., and Li, X. (2011). Transcriptional regulation of mammalian miRNA genes. Genomics *97*, 1–6.
4. Lee, Y., Ahn, C., Han, J., Choi, H., Kim, J., Yim, J., Lee, J., Provost, P., Rådmark, O., Kim, S., and Kim, V.N. (2003). The nuclear RNase III Drosha initiates microRNA processing. Nature *425*, 415–419.
5. Pillai, R.S., Artus, C.G., and Filipowicz, W. (2004). Tethering of human Ago proteins to mRNA mimics the miRNA-mediated repression of protein synthesis. RNA *10*, 1518–1525.
6. Park, J.H., and Shin, C. (2014). MicroRNA-directed cleavage of targets: mechanism and experimental approaches. BMB Rep. *47*, 417–423.
7. Lewis, B.P., Burge, C.B., and Bartel, D.P. (2005). Conserved seed pairing, often flanked by adenosines, indicates that thousands of human genes are microRNA targets. Cell *120*, 15–20.

8. Iwakawa, H.O., and Tomari, Y. (2015). The Functions of MicroRNAs: mRNA Decay and Translational Repression. Trends Cell Biol. *25*, 651–665.

9. Hayes, J., Peruzzi, P.P., and Lawler, S. (2014). MicroRNAs in cancer: biomarkers, functions and therapy. Trends Mol. Med. *20*, 460–469.

10. Beg, M.S., Brenner, A.J., Sachdev, J., Borad, M., Kang, Y.K., Stoudemire, J., Smith, S., Bader, A.G., Kim, S., and Hong, D.S. (2017). Phase I study of MRX34, a liposomal miR-34a mimic, administered twice weekly in patients with advanced solid tumors. Invest. New Drugs *35*, 180–188.

11. Chen, F., and Hu, S.-J. (2012). Effect of microRNA-34a in cell cycle, differentiation, and apoptosis: a review. J. Biochem. Mol. Toxicol. *26*, 79–86.

12. Misso, G., Di Martino, M.T., De Rosa, G., Farooqi, A.A., Lombardi, A., Campani, V., Zarone, M.R., Gullà, A., Tagliaferri, P., Tassone, P., and Caraglia, M. (2014). Mir-34: a new weapon against cancer? Mol. Ther. Nucleic Acids *3*, e194.

13. Li, X.J., Ren, Z.J., and Tang, J.H. (2014). MicroRNA-34a: a potential therapeutic target in human cancer. Cell Death Dis. *5*, e1327.

14. Khvorova, A., Reynolds, A., and Jayasena, S.D. (2003). Functional siRNAs and miRNAs exhibit strand bias. Cell *115*, 209–216.

15. Guennewig, B., Roos, M., Dogar, A.M., Gebert, L.F., Zagalak, J.A., Vongrad, V., Metzner, K.J., and Hall, J. (2014). Synthetic pre-microRNAs reveal dual-strand activity of miR-34a on TNF-α. RNA *20*, 61–75.

16. O'Toole, A.S., Miller, S., and Serra, M.J. (2005). Stability of 3′ double nucleotide overhangs that model the 3′ ends of siRNA. RNA *11*, 512–516.

17. Gao, J., Li, N., Dong, Y., Li, S., Xu, L., Li, X., Li, Y., Li, Z., Ng, S.S., Sung, J.J., et al. (2015). miR-34a-5p suppresses colorectal cancer metastasis and predicts recurrence in patients with stage II/III colorectal cancer. Oncogene *34*, 4142–4152.

18. Fadok, V.A., Bratton, D.L., Frasch, S.C., Warner, M.L., and Henson, P.M. (1998). The role of phosphatidylserine in recognition of apoptotic cells by phagocytes. Cell Death Differ. *5*, 551–562.

19. Raver-Shapira, N., Marciano, E., Meiri, E., Spector, Y., Rosenfeld, N., Moskovits, N., Bentwich, Z., and Oren, M. (2007). Transcriptional activation of miR-34a contributes to p53-mediated apoptosis. Mol. Cell *26*, 731–743.

20. Clark, A.G., and Vignjevic, D.M. (2015). Modes of cancer cell invasion and the role of the microenvironment. Curr. Opin. Cell Biol. *36*, 13–22.

21. Wang, G., Liu, G., Ye, Y., Fu, Y., and Zhang, X. (2016). Upregulation of miR-34a by diallyl disulfide suppresses invasion and induces apoptosis in SGC-7901 cells through inhibition of the PI3K/Akt signaling pathway. Oncol. Lett. *11*, 2661–2667.

22. Boon, R.A., Iekushi, K., Lechner, S., Seeger, T., Fischer, A., Heydt, S., Kaluza, D., Tréguer, K., Carmona, G., Bonauer, A., et al. (2013). MicroRNA-34a regulates cardiac ageing and function. Nature *495*, 107–110.

23. Paidikondala, M., Rangasami, V.K., Nawale, G.N., Casalini, T., Perale, G., Kadekar, S., Mohanty, G., Salminen, T., Oommen, O.P., and Varghese, O.P. (2019). An Unexpected Role of Hyaluronic Acid in Trafficking siRNA Across the Cellular Barrier: The First Biomimetic, Anionic, Non-Viral Transfection Method. Angew. Chem. Int. Ed. Engl. *58*, 2815–2819.

24. Jin, H.Y., Gonzalez-Martin, A., Miletic, A.V., Lai, M., Knight, S., Sabouri-Ghomi, M., Head, S.R., Macauley, M.S., Rickert, R.C., and Xiao, C. (2015). Transfection of microRNA Mimics Should Be Used with Caution. Front. Genet. *6*, 340.

25. Søkilde, R., Newie, I., Persson, H., Borg, Å., and Rovira, C. (2015). Passenger strand loading in overexpression experiments using microRNA mimics. RNA Biol. *12*, 787–791.

26. Kim, Y.K., Heo, I., and Kim, V.N. (2010). Modifications of small RNAs and their associated proteins. Cell *143*, 703–709.

27. Liu, C., Kelnar, K., Liu, B., Chen, X., Calhoun-Davis, T., Li, H., Patrawala, L., Yan, H., Jeter, C., Honorio, S., et al. (2011). The microRNA miR-34a inhibits prostate cancer stem cells and metastasis by directly repressing CD44. Nat. Med. *17*, 211–215.

28. Krzeszinski, J.Y., Wei, W., Huynh, H., Jin, Z., Wang, X., Chang, T.C., Xie, X.J., He, L., Mangala, L.S., Lopez-Berestein, G., et al. (2014). miR-34a blocks osteoporosis and bone metastasis by inhibiting osteoclastogenesis and Tgif2. Nature *512*, 431–435.

OMTN, Volume *16*

Supplemental Information

Synthetic Design of Asymmetric miRNA with an Engineered 3′ Overhang to Improve Strand Selection

Sandeep Kadekar, Ganesh N. Nawale, Kira Karlsson, Cecilia Ålander, Oommen P. Oommen, and Oommen P. Varghese

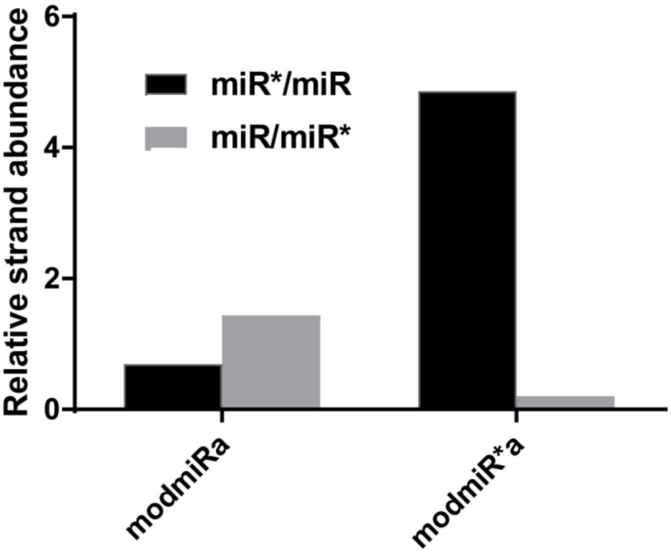

Figure S1. Stem loop qPCR analysis of strand recruitment for 5-nucleotide overhang miRNAs (modmiRa and modmiR*).

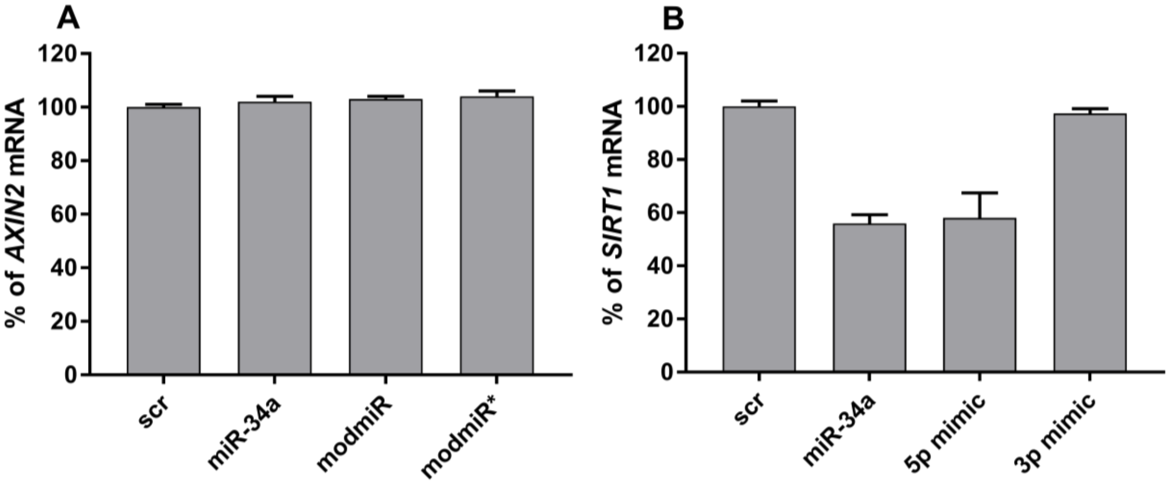

Figure S2. qPCR analysis for A) *AXIN2* and B) *SIRT1* mRNA levels. Cells were treated with different miRNAs for 48 h.

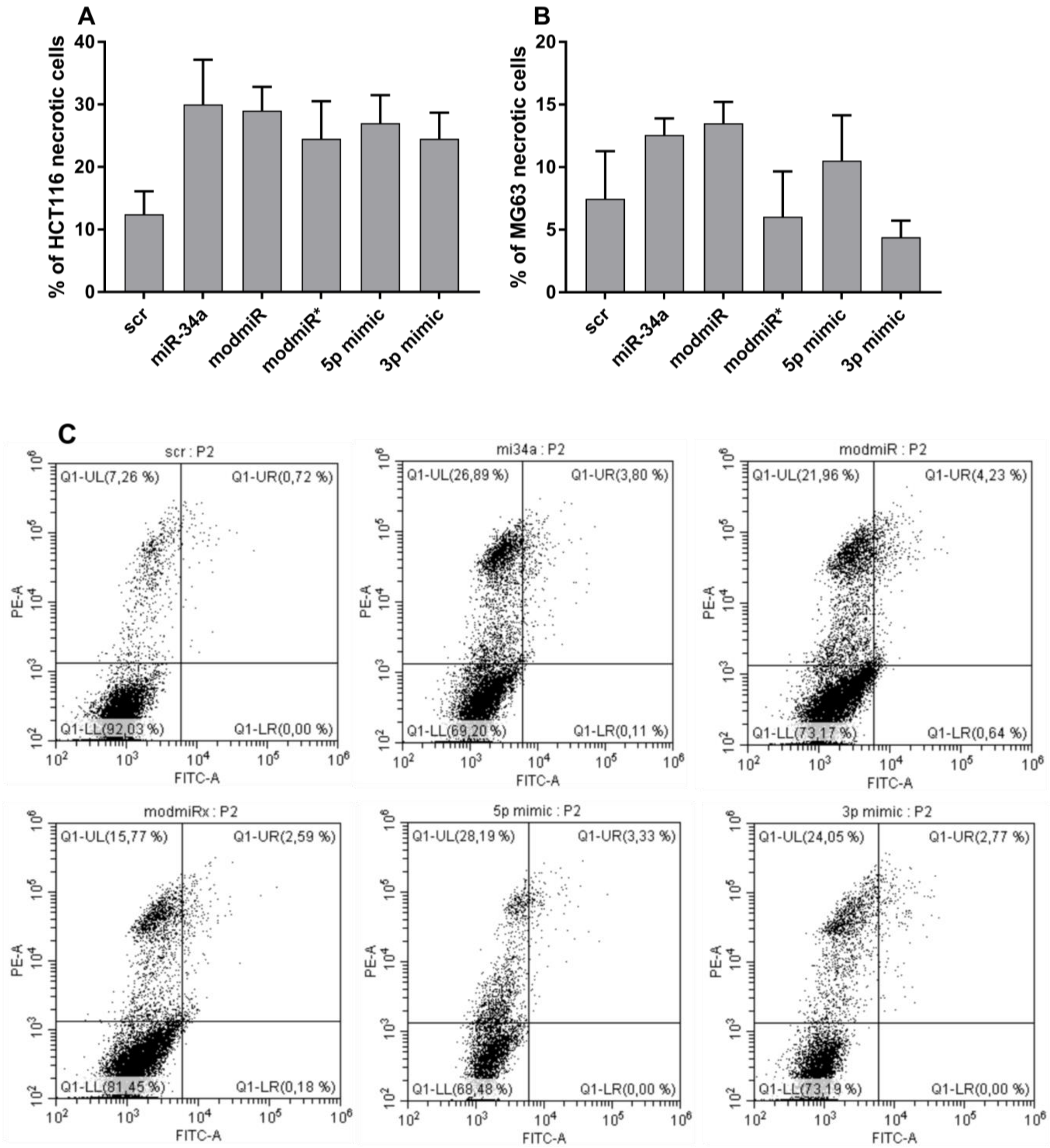

Figure S3. FACS analysis for HCT116 and MG63 cells. Cells were treated with different miRNAs for 48 h. A) and C) HCT116 cells were analysed for apoptotic and necrotic cells. B) MG63 cells were analysed for necrotic cells.
